# Serum thiamin concentration is negatively correlated with lactate levels in survivors of septic shock

**DOI:** 10.1186/cc12671

**Published:** 2013-06-19

**Authors:** NA Costa, MS Dorna, AL Gut, PS Azevedo, LAM Zornoff, SAR Paiva, MF Minicucci

**Affiliations:** 1 Botucatu Medical School, UNESP, Rubião Júnior, Botucatu, SP, Brazil

## Introduction

Thiamine deficiency can be present in 20% of patients in the ICU. This deficiency is considered to be an uncommon source of lactic acidosis in septic patients. An elevated serum lactate level is associated with morbidity and mortality [1]. Increased levels of thiamine increase the activity of glutathione peroxidase (GPx), a major component of the cellular antioxidant system [2]. The objective of this study was to determine the influence of serum thiamine concentrations on lactate levels, GPx activity, length of hospital stay, length of ICU stay and ICU mortality in patients with septic shock.

## Methods

This prospective study included all patients with septic shock on admission or during ICU stay, over the age of 18, admitted to one of the three ICUs of the Botucatu Medical School from January to August 2012. Demographic information, clinical evaluation and blood samples were taken within the first 72 hours of the patient's admission or within 72 hours after septic shock diagnosis for laboratory analysis, serum thiamine and GPx activity determination. The level of significance was set at 5%.

## Results

One hundred and eight consecutive patients were evaluated. The mean age was 57.5 ± 16.0 years, 63% were male and 54.6% died in the ICU. The frequency of thiamine deficiency was 71.3%. Neither the serum thiamine concentration nor the erythrocyte GPx activity was associated with mortality in septic shock patients. Thiamine levels were also not associated with GPx activity (*r *= 0.141, *P *= 0.165). In addition, thiamine levels were not associated with serum lactate in the 108 patients with septic shock (*r *= -0.074, *P *= 0.444). However, vitamin B1 levels were negatively associated with lactate in patients who survived (*r *= -0.311, *P *= 0.029). In the regression model analysis, vitamin B1 levels were not associated with ICU mortality or with the length of the ICU or hospital stay.

## Conclusion

Thiamine deficiency is common in septic shock patients. Furthermore, thiamine was negatively associated with lactate levels in survivors of septic shock.
